# The mitochondrial UPR induced by ATF5 attenuates intervertebral disc degeneration via cooperating with mitophagy

**DOI:** 10.1007/s10565-024-09854-9

**Published:** 2024-03-13

**Authors:** Wen-Ning Xu, Huo-Liang Zheng, Run-Ze Yang, Yuan-Fang Sun, Bi-Rong Peng, Chun Liu, Jian Song, Sheng-Dan Jiang, Li-Xin Zhu

**Affiliations:** 1 Department of Spinal Surgery, Orthopedic Medical Center, Zhujiang Hospital, Southern Medical University, Guangzhou, 510280 China; 2 Department of Clinic of Spine Center, Xinhua Hospital, Shanghai Jiaotong University School of Medicine, Shanghai, 200082 China; 3 Department of Orthopedics, Orthopedic Research Institute, West China Hospital, Sichuan University, Chengdu, China; 4 Department of Orthopedics, Huashan Hospital Fudan University, Shanghai, 200040 China

**Keywords:** Intervertebral disc degeneration, Mitochondrial unfolded protein response, Atf5, Pink1, Mitophagy

## Abstract

Intervertebral disc degeneration (IVDD) is an aging disease that results in a low quality of life and heavy socioeconomic burden. The mitochondrial unfolded protein response (UPR^mt^) take part in various aging-related diseases. Our research intents to explore the role and underlying mechanism of UPR^mt^ in IVDD. Nucleus pulposus (NP) cells were exposed to IL-1β and nicotinamide riboside (NR) served as UPR^mt^ inducer to treat NP cells. Detection of ATP, NAD + and NADH were used to determine the function of mitochondria. MRI, Safranin O-fast green and Immunohistochemical examination were used to determine the degree of IVDD in vivo. In this study, we discovered that UPR^mt^ was increased markedly in the NP cells of human IVDD tissues than in healthy controls. In vitro, UPR^mt^ and mitophagy levels were promoted in NP cells treated with IL-1β. Upregulation of UPR^mt^ by NR and Atf5 overexpression inhibited NP cell apoptosis and further improved mitophagy. Silencing of Pink1 reversed the protective effects of NR and inhibited mitophagy induced by the UPR^mt^. In vivo, NR might attenuate the degree of IDD by activating the UPR^mt^ in rats. In summary, the UPR^mt^ was involved in IVDD by regulating Pink1-induced mitophagy. Mitophagy induced by the UPR^mt^ might be a latent treated target for IVDD.

**Graphical Abstract:**

• UPR^mt^ was upregulated in the NP cells of degenerative intervertebral disc.

• UPR^mt^ regulated by Atf5 could activate mitophagy to protect NP cells from apoptosis.

• Nicotinamide riboside as UPR^mt^ inducer reduced NP cells apoptosis, thereby delaying the process of IVDD.

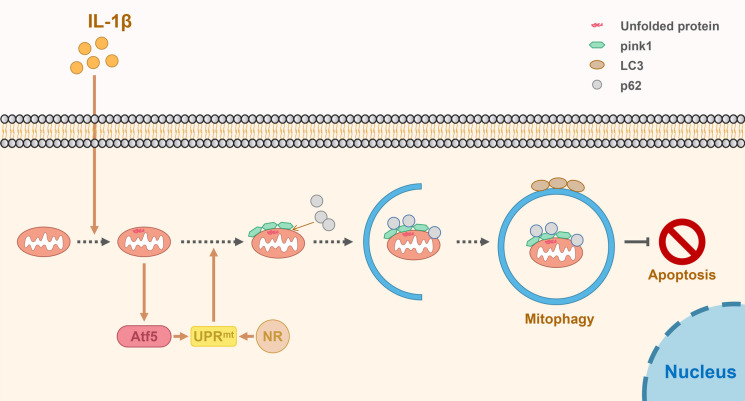

**Supplementary Information:**

The online version contains supplementary material available at 10.1007/s10565-024-09854-9.

## Introduction

Intervertebral disc degeneration (IVDD), as a prevailing and multifactorial disorder in orthopedics, is the main cause of low back pain (Xu et al. 2019a, b). To date, the clinical treatment strategies for diseases such as disc degeneration are mainly limited to surgery (Dowdell et al. 2017). Direct intervention for IVDD in the early stage of disease is the most direct and minimally invasive method. Therefore, it is important to investigate the mechanism of IVDD.

Mitochondria are important organelles for biological oxidation and energy conversion in eukaryotic cells (Hou et al. 2019). The response in which mitochondrial dysfunction leads to the accumulation of large amounts of unfolded proteins and induces changes in gene transcription and cytoactive is called the mitochondrial unfolded protein response (UPR^mt^) (Shpilka and Haynes 2017; Jovaisaite and Auwerx 2014). Mitochondrial dysfunction leads to accumulation of large numbers of unfolded proteins in the mitochondria, which induces nuclear encoding mitochondrial chaperone proteins and proteases, such as heat shock protein family A member 1A (HSPA1A), heat shock protein family A member 9 (HSPA9), heat shock protein family E (Hsp10) member 1 (HSPE1), YME1-like 1 ATPase (YME1-like 1 ATPase, YME1L1), caseinolytic mitochondrial matrix peptidase proteolytic subunit (CLPP), mitochondrial lon peptidase 1 (LONP1) and other proteins, which are upregulated to improve mitochondrial function (Zhang et al. 2018; Melber and Haynes 2018) and delaying aging (Zhang et al. 2016). The UPR^mt^ is involved in Parkinson's disease, Alzheimer's disease, hereditary spastic paraplegia, and Friedreich ataxia and other aging-related diseases (Shpilka and Haynes 2017; Mena et al. 1996; Guillon et al. 2009). However, as a classic aging-related disease, the role of UPR^mt^ in IVDD still lacks systematic research.

Mitophagy, one type of substance-specific autophagy, mediates the selective removal of mitochondria and has been widely studied. Early studies have shown that mitophagy adapts to metabolic needs and quality control by regulating the number of mitochondria and removing damaged mitochondria (Tolkovsky et al. 2002). The mitophagy signaling pathway induced by PINK1 and Parkin plays a keyrole in mitophagy in mammalian cells. Abnormal regulation of Parkin is related to Parkinson's disease and the loss of substantia nigra neurons. Activation of PINK1 is required for Parkin to decouple mitochondrial translocation to induce mitophagy. In fact, in the case of mitochondrial uncoupling, experimentally induced PINK1 accumulation or ectopic expression of PINK1 can induce parkin translocation and mitophagy (Clark et al. 2006; Park et al. 2006). Despite a major novel observation about PINK1 of expression and import in mitochondria was found, PINK1 is expeditious developed into degradation condition by means of proteolysis and then is kept extremely low. When damaged mitochondria lead to inhibition of PINK1 proteolysis, PINK1 aggregates in damaged mitochondria, and Parkin is subsequently recruited by accumulated PINK1 (Matsuda et al. 2010). The occurrence of mitophagy is related to Parkin-mediated ubiquitination of mitochondrial substrates.

In summary, this study hypothesis that under pathological conditions, the UPR^mt^ induced mitophagy by Pink1 to protect nucleus pulposus (NP) cells from apoptosis, enhance antioxidant function and promote mitochondrial function in NP cells. The enhanced UPR^mt^ in intervertebral disc cells may attenuate IVDD via mitophagy in vivo.

## Materials and methods

### Patients and samples

The sample size and collection methods for the human were merely a convenience according to previous study (Cheng et al. 2018; Xu et al. 2023). Patient information is listed in Table S1. The above patients signed informed consent forms. This study was approved by the ethics committee of Xinhua Hospital Affiliated to Shanghai Jiaotong University School of Medicine, and all protocols were performed according to the Declaration of Helsinki.

### Cell culture

NP cells were isolated from the rat lumbar intervertebral discs (Risbud et al. 2005). Briefly, male SD rats (6 weeks old) were euthanized by CO_2_. The spine was removed under sterile conditions and the lumbar disc was gained. Gelatinous NPs were isolated from the fibrous loop under a dissecting microscope and then treated with collagenase for 3 h. The nucleus pulposus tissue after centrifugation was transferred to DMEM/F12 medium supplemented with 10% FBS. After 2 weeks of cell confluency, primary cells were collected with 0.25% trypsin–EDTA and transplanted into suitable culture plates. Second-generation cells were used for the follow-up experiment.

### Transfection of small interfering RNA

Small interfering RNAs (GenePharma, Shanghai, China) for Pink1, Atf5 and overexpressed plasmid for Atf5 (si-Pink1, si-Atf5 and OE-Atf5) were designed according to previous studies (Angelastro et al. 2005; Huang et al. 2017). The sequences of si-Pink1 and si-Atf5 are listed in Table S2. Briefly, siRNAs and Lipofectamine 3000 (Invitrogen, Carlsbad, California, CA) were mixed in Opti-MEM medium (Thermo Fisher, USA) for 20 min. Then the mixtures were transferred to medium.

### Lentivirus transfection

When NP cells in the six-well plate grow to 80% confluency, we added polybrene and 1 μM of mRFP-GFP-LC-3. After 48 h of incubation, follow-up experiments were carried out to detect autophagy flux. Green dots indicate initial autophagosomes, and red dots indicate autophagolysosomes.

### Immunofluorescence examination

NP cells were seeded on glass slides in 60 mm plates. We fixed NP cells with 4% paraformaldehyde. 0.3% Triton X-100 penetrated cells for 15 mim.. 5% BSA block NP cells. Primary antibodies were added to the glass slides and incubated overnight at 4 °C. Pink1 rabbit polyclonal antibody (Proteintech, Cat. No. 23274–1-AP, 1:100, USA) and Hspd1 mouse polyclonal antibody (Santa Cruz, Cat. No. sc-59567, 1:100, USA) were incubated together. Hspa1a rabbit polyclonal antibody (Proteintech, Cat. No. 10995–1-AP, 1:100, USA) and Clpp mouse polyclonal antibody (Santa Cruz, Cat. No. sc-271284, 1:100, USA) were incubated together. Lonp1 rabbit polyclonal antibody (Proteintech, Cat No. 15440–1-AP, 1:500, USA) and Clpp mouse polyclonal antibody (Santa Cruz, Cat. No. sc-271284, 1:100, USA) were incubated together. Subsequently, Alexa Fluor 488-conjugated AffiniPure goat anti-mouse IgG (H + L) and Cy3-conjugated AffiniPure goat anti-rabbit IgG (H + L) were added to glass slides and the incubation.

### Quantitative real-time PCR (qRT-PCR)

QRT-PCR referred to our previous study (Xu et al. 2023). The specific primer sequences were listed in Table S3.

### Western blot analysis

The extraction of total protein and mitochondrial protein was performed according to a previous study (Xu et al. 2019a, 2023). Proteins were electroblotted onto polyvinylidene difluoride membranes. We blocked the membrane containing the protein of interest for 2 h using 5% evaporated milk and bathed with the primary antibody overnight at 4 °C. The information of primary antibodies was in Table S4. The corresponding horseradish peroxidase-conjugated secondary antibody integrated membrane. Membranes of the protein were luminescent using ECL plus reagent (Millipore) on the ChemiDocTM XRS + system (Bio-Rad, USA).

### Flow cytometry assays

After performing IL-1β stimulation, 5 μl of Annexin V-FITC and 10 μl of PI (BD, Cat. No. 556547, USA) were used to measure NP cell apoptosis according to our previous study (Xu et al. 2019a).

### TUNEL assays for apoptosis

After performing IL-1β stimulation, 4% paraformaldehyde fixed NP cells. 0.3% Triton X-100 penetrated cells at room temperature for 5 min. The sample was added with 50 μl TUNEL solution and incubated at 37℃ for 60 min without light. Nuclei were stained with DAPI. Fluorescence microscope was observed after sealing with anti-fluorescence quenching sealing solution.

### NAD^+^ and NADH quantification

Approximately 5,000–10,000 NP cells were obtained. We directly sorted lysate buffer provided by the NAD + /NADH detection kit (BioVision, k337-100) into the cells. Then, the NAD + and NADH concentrations were determined by spectrophotometer, and the detection volume was reduced by 50% in all steps. The result was computed from the standard curve generated from the NADH standard product in the kit.

### ATP quantification

After performing apoptosis stimulation, 200 μl of lysate from the ATP detection kit (Beyotime, Cat. No. S0026, China) was added to the plates, and NP cells were completely lysed by using a pipette to repeatedly blow to make the lysate fully contact and lyse the cells. After lysis, the supernatant was centrifuged at 12000 g at 4 °C for 5 min and used for subsequent determination. A standard curve was made according to the instructions. Then, a luminometer was used to determine the RLU (luminescence) value. Finally, the concentration of ATP in the sample was computed based on standard curve.

### Magnetic resonance imaging method

7.0 T clinical magnet (Philips Intera Achieva 7.0MR) was used to obtain sagittal T2-weighted images. Changes in T2 signal and structure were evaluated by a blinded orthopedic researcher using the classification of IVDD reported by Pfirrmann et al. (Pfirrmann et al. 2001) (grade I = 1 point, grade II = 2 points, grade III = 3 points, grade IV = 4 points, grade V = 5 points).

### Safranin O-fast green staining

The methods of Safranin O-fast green staining refered to our previous research (Xu et al. 2019a).

### Immunohistochemical examination

The sections were prepared already. The procedure was based on our previous study (Xu et al. 2023). The information of primary antibodies were listed: HSPA1A (Abcam, Cat. No. ab181606, 1:50, UK), HSPD1 (Proteintech, Cat. No. 15282–1-AP, 1:200, USA), Clpp (Proteintech, Cat. No. 15440–1-AP, 1:200, USA), Lonp1 (Proteintech, Cat. No. 15698–1-AP, 1:200, USA), p21 (Proteintech, Cat. No. 28248–1-AP, 1:50, USA) and p53 (Proteintech, Cat. No. 10442–1-AP, 1:50, USA). HRP-conjugated secondary antibody (Santa Cruz Biotechnology, Dallas, TX, USA) was added to sections. Morphology were taken by Image-Pro Plus software version 6.0 (Media Cybernetics, Rockville, MD, USA).

### Surgical procedure

We purchased eight-week-old healthy male adult Sprague‒Dawley rats from Shanghai SLAC Laboratory Animal Co., Ltd. The sample size ensured at least three replicate experiments. All animals were fed in the SPF animal room of Xinhua Hospital Affiliated with Shanghai Jiao Tong University School of Medicine. Disc degenerative models were established with Sprague‒Dawley rats. All experiments were performed in accordance with the International Guiding Principles for Biomedical Research Involving Animals and approved by the Ethics Committee of Xinhua Hospital Affiliated with Shanghai Jiao Tong University School of Medicine. 24 8-week-old healthy male adult rats were randomly divided into four groups according to the lottery and the experimental unit was a single animal: sham operation group, sham operation + NR group, IVDD group and IVDD + NR group. According to previous study, pellets supplemented a loading agent or NR (400 mg/kg/day) for 2 months was used to fed rats (Li et al. 2016). h. The surgical procedure was carried out based on our previous study (Xu et al. 2019a). Two months after surgery, 6 rats in each group were assessed by micro-MRI. Rat was performed by humanitarian executions. The disc tissues of rats were obtained. Rat intervertebral disc of L1-2, L2-3, L3-4, L4-5 and L5-6 were used to perform in follow-up experiments. Each rat included these discs were assessed by Micro-MRI. The Pfirrmann score of intervertebral discs were assessed in a double-blind manner. Intervertebral disc from the same segment were used in the same experiment were used to perform the same.

### Statistical analysis

The data are presented as the mean ± SD (standard deviation) with confidence interval (CI). GraphPad Prism 8 were used to analyze the statistic of data. One-way ANOVA and Tukey’s post-hoc test was used to analyze multiple comparisons of data. Independent-samples t tests were used The differences between two groups were analyzed by independent-samples. In vivo, two-way ANOVA and Holm-Sidak's multiple comparisons test was used to analyze multiple comparisons of data among the groups. MRI Pfirrmann greades of rat intervertebral discs was analyzed by Kruskall-Wallis. Averaging prior to analysis were performed. The *p* value < 0.05 was considered to be statistically significant. ***p < 0.001, **p < 0.01, *p < 0.05. All experiments were performed at least three times.

## Results

### The UPR^mt^ is decreased in NP cells of IVDD tissue

**Fig. 1:**
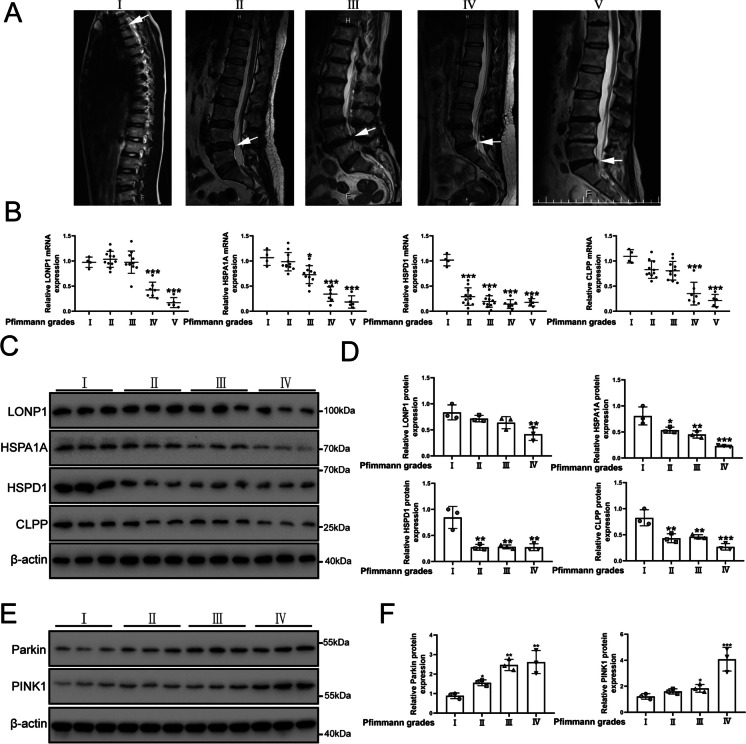
The expression of the UPR^mt^ marker genes was reduced in the human IVDD tissue samples and increased in NP cells after IL-1β treatment. (**A**) The representing graphics of each Pfirrman grade was shown. The specific position of the obtained intervertebral disc tissue was indicated by arrows. (**B**) the UPR^mt^ markers including LONP1, HSPA1A, HSPD1 and CLPP in the human IVDD tissue samples were detected by qRT-PCR. Pfirrman grade I (*n* = 4), Pfirrman grade II (*n* = 11), Pfirrman grade III (*n* = 11), Pfirrman grade IV (*n* = 8) and Pfirrman grade I (*n* = 5). (**C**) Western blot to the UPR^mt^ including LONP1, HSPA1A, HSPD1 and CLPP in the human IVDD tissue samples. Pfirrman grade I (*n* = 3), Pfirrman grade II (*n* = 3), Pfirrman grade III (*n* = 3) and Pfirrman grade IV (*n* = 3). (**D**) The quantitative analysis to the results of Western blot. (**E**) Western blot to mitophagy markers including Parkin, Pink1 and β-actin in the human IVDD tissue samples. Pfirrman grade I (*n* = 3), Pfirrman grade II (*n* = 3), Pfirrman grade III (*n* = 3) and Pfirrman grade IV (*n* = 3). (**F**) The quantitative analysis to the results of Western blot. Statistical significance was analyzed by one-way ANOVA followed by a post hoc Tukey’s test. All data were presented as mean ± SD. * *p* < 0.05; ** *p* < 0.01; *** *p* < 0.001

MRI imaging grading for IVDD was according to the Pfirrmann scoring system (Pfirrmann et al. 2001). The origin of human intervertebral disc tissues had been described in previous study (Stirling et al. 2001). Representative MRI of the patients was shown in Fig. 1A. We found that the mRNA expression of CLPP (named Clpp in rats), HSPA1A (named Hspa1a in rats), HSPD1 (named Hspd1 in rats) and LONP1 (named Lonp1 in rats), well-known markers of the UPR^mt^, was decreased in human IVDD tissue samples (Fig. 1B). The protein expression of CLPP, HSPA1A, HSPD1 and LONP1 decreased significantly with an increase in the degree of IVDD (Fig. 1C and D). However, the results of Immunohistochemical examination showed that HSPA1A and HSPD1 protein expression was increased in the NP cells of human IVDD tissues (Fig. S1A). These results showed that the UPR^mt^ process were varied differently in different cells during disc degeneration and that the UPR^mt^ might be involved in the process of IVDD. Interestingly, Parkin and PINK1 (named Pink1 in rats) protein expression was increased in the human IVDD tissue samples (Fig. 1E and F). The results were contrary to those of the UPR^mt^ in human disc degeneration. However, the underlying mechanism between UPR^mt^ and mitophagy remains unclear.

**Fig. 2:**
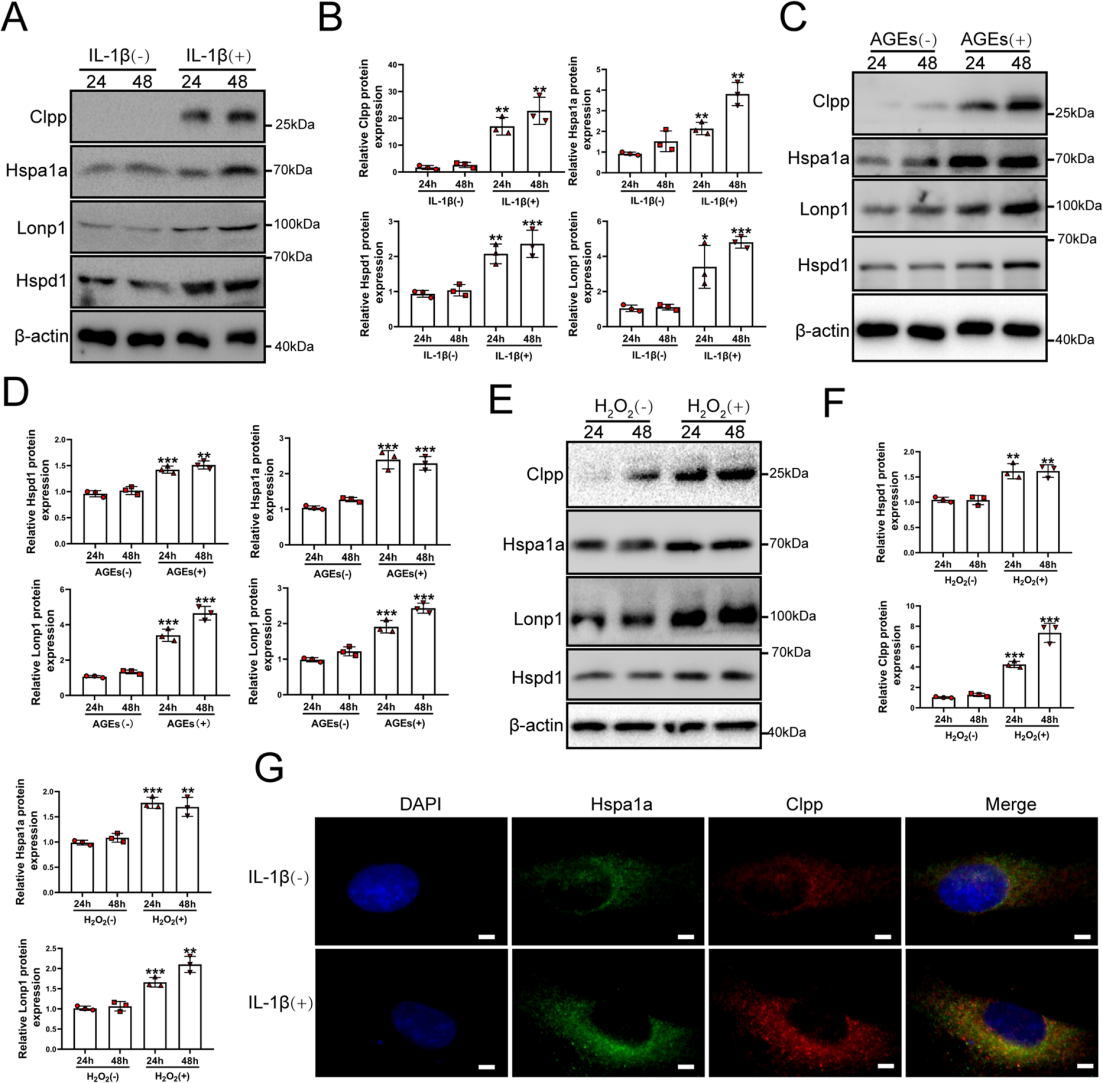
The expression of the UPR^mt^ marker genes was also increased in NP cells after stress. (**A**-**B**) NP cells were treated with Vehicle or IL-1β (20 ng/ml) for 24 h and 48 h. (**A**) Western blot to the UPR^mt^ markers such as Clpp, Hspa1a, Lonp1, Hspa9, Hspd1 and β-actin in NP cells after IL-1β treatment (*n* = 3). (**B**) The quantitative analysis to the results of Western blot. (**C**) Western blot to the UPR^mt^ markers such as Hspd1, Hspa1a, Clpp, Lonp1, and β-actin in NP cells (*n* = 3). NP cells were treated with Vehicle or advanced glycation end products (AGEs, 200 μg/mL) for 24 h and 48 h. (**D**) The quantitative analysis to the results of Western blot. (**E**) Western blot to the UPR^mt^ markers such as Hspd1, Hspa1a, Clpp, Lonp1, and β-actin in NP cells (*n* = 3). NP cells were treated with Vehicle or hydrogen peroxide (H_2_O_2_, 100 μM) for 24 h and 48 h. (**F**) The quantitative analysis to the results of Western blot. (**G**) Immunofluorescence analysis to the UPR^mt^ makers including Hspa1a and Clpp in NP cells after IL-1β treatment (*n* = 3). NP cells were treated with Vehicle or IL-1β (20 ng/ml) for 48 h. Scale bars = 5 μm. Statistical significance was analyzed by one-way ANOVA followed by a post hoc Tukey’s test. All data were presented as mean ± SD. *p < 0.05; **p < 0.01; ***p < 0.001

To investigate the underlying mechanism of the UPR^mt^ in IVDD, we isolated NP cells from rats and performed the following experiments in NP cells in vitro. Interleukin (IL)-1β, advanced glycation end products and oxidative stress are classical factor that led to NP cell senescence and apoptosis, resulting in IVDD (Liao et al. 2019; Wang et al. 2019; Yang et al. 2022; Zhang et al. 2022; Lin et al. 2021; Cheng et al. 2021; Chen et al. 2020). According to a previous study (Luo et al. 2021), NP cells were exposed to 20 ng/ml IL-1β for 24 and 48 h. Mitochondrial dysfunction leads to oxidative stress, apoptosis and premature cell senescence, which are all related to IVDD (Saberi et al. 2021; Zhang et al. 2020). mRNA and protein expression of UPR^mt^ markers, including Clpp, Hspa1a, Hspd1 and Lonp1 was increased by IL-1β (Fig. S2A and Fig. 2A). Increased Hspa1a and Clpp protein expression was confirmed in NP cells by immunofluorescence assays (Fig. 2G). After IL-1β treatment, we determined mitochondrial dysfunction in NP cells by the reduced expression of TCA cycle and OXPHOS genes (Fig. S2B). As was shown in Fig. 2, advanced glycation end products and hydrogen peroxide (H_2_O_2_) treatments caused an increased UPR^mt^ process (Fig. 2C-F). These results showed that stress could induce the UPR^mt^ in NP cells.

### Nicotinamide riboside activates the UPR^mt^ in NP cells after IL-1β treatment

**Fig. 3:**
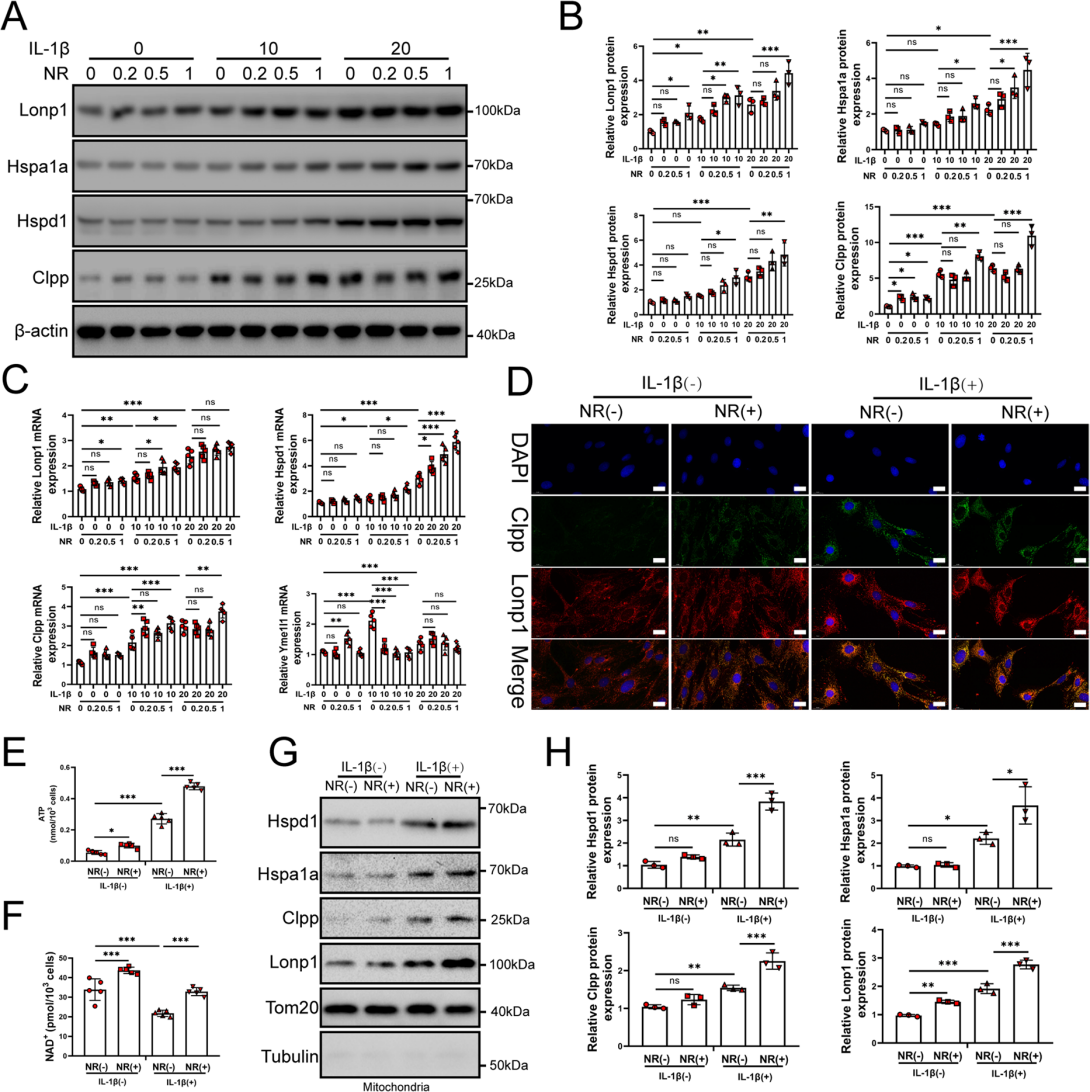
NR promote the level of the UPR^mt^ in NP cells. (A-C) NP cells were divided into 3 groups and treated with 0 ng/ml, 10 ng/ml and 20 ng/ml IL-1β for 48 h respectively. Each group was subdivided into 4 groups and treated with 0, 0.2, 0.5 and 1 mM NR for 6 h respectively after IL-1β treatments. (**A**) Western blot to the UPR^mt^ markers such as Lonp1, Hspa4, Hspd1 and Clpp in NP cells (*n* = 3). (**B**) The quantitative analysis to the results of Western blot. (**C**) the UPR^mt^ makers such as Lonp1, Hspa4, Hspd1 and Clpp in NP cells were detected by qRT-PCR (*n* = 5). (**D**) Immunofluorescence analysis to the UPR^mt^ makers including Hspa4 and Hspa9 in NP cells after IL-1β treatment (*n* = 3). NP cells were divided into 2 groups and treated with 0 ng/ml and 20 ng/ml IL-1β for 48 h respectively. Each group was subdivided into 2 groups and treated with 0 and 1 mM NR for 6 h respectively after IL-1β treatments. Scale bars = 20 μm. (**E**) Cellular ATP level (**F**) NAD^+^ concentrations in NP cells (*n* = 5). NP cells were divided into 2 groups and treated with 0 ng/ml and 20 ng/ml IL-1β for 48 h respectively. Each group was subdivided into 2 groups and treated with 0 and 1 mM NR for 6 h respectively after IL-1β treatments. (**G**) The relative expression of Parkin, Pink1, Sqstm1, Clpp, Lonp1 and Hspd1 were determined by Western blot in the mitochondria of NP cells (*n* = 3). NP cells were divided into 2 groups and treated with 0 ng/ml and 20 ng/ml IL-1β for 48 h respectively. Each group was subdivided into 2 groups and treated with 0 and 1 mM NR for 6 h respectively after IL-1β treatments. The experimental grouping settings were indicated. (**H**) The quantitative analysis to the results of Western blot. Statistical significance was analyzed by one-way ANOVA followed by a post hoc Tukey’s test. All data were presented as mean ± SD. *p < 0.05; **p < 0.01; ***p < 0.001

Nicotinamide riboside (NR), an NAD^+^-boosting compound, was considered to improve the level of the UPR^mt^, which delays aging in mice (Zhang et al. 2016; Sorrentino et al. 2017). To date, the role of NR-induced UPR^mt^ has not been evaluated in IVDD. Therefore, we treated NP cells with 0.2, 0.5 and 1 mM of NR for 6 h after pretreating IL-1β (10 and 20 ng/ml). As was shown in Fig. 3A, B and C, along with the increased concentration of NR, there was increased mRNA and protein expression of Lonp1, Hspa1a, Hspd1 and Clpp but not Yme1l1 (Fig. 3A-C). Immunofluorescence results also confirmed that NR further caused increased expression of Lonp1 and Clpp in NP cells (Fig. 3D). NR significantly improved the mitochondrial function of NP cells, as shown by an increase in ATP and NAD^+^ levels (Fig. 3E and F). Increased UPR^mt^ markers also were observed in mitochondria (Fig. 3G and H). These results demonstrated that NR could induce UPR^mt^.

### NR promotes the survival of NP cells via the UPR^mt^

**Fig. 4:**
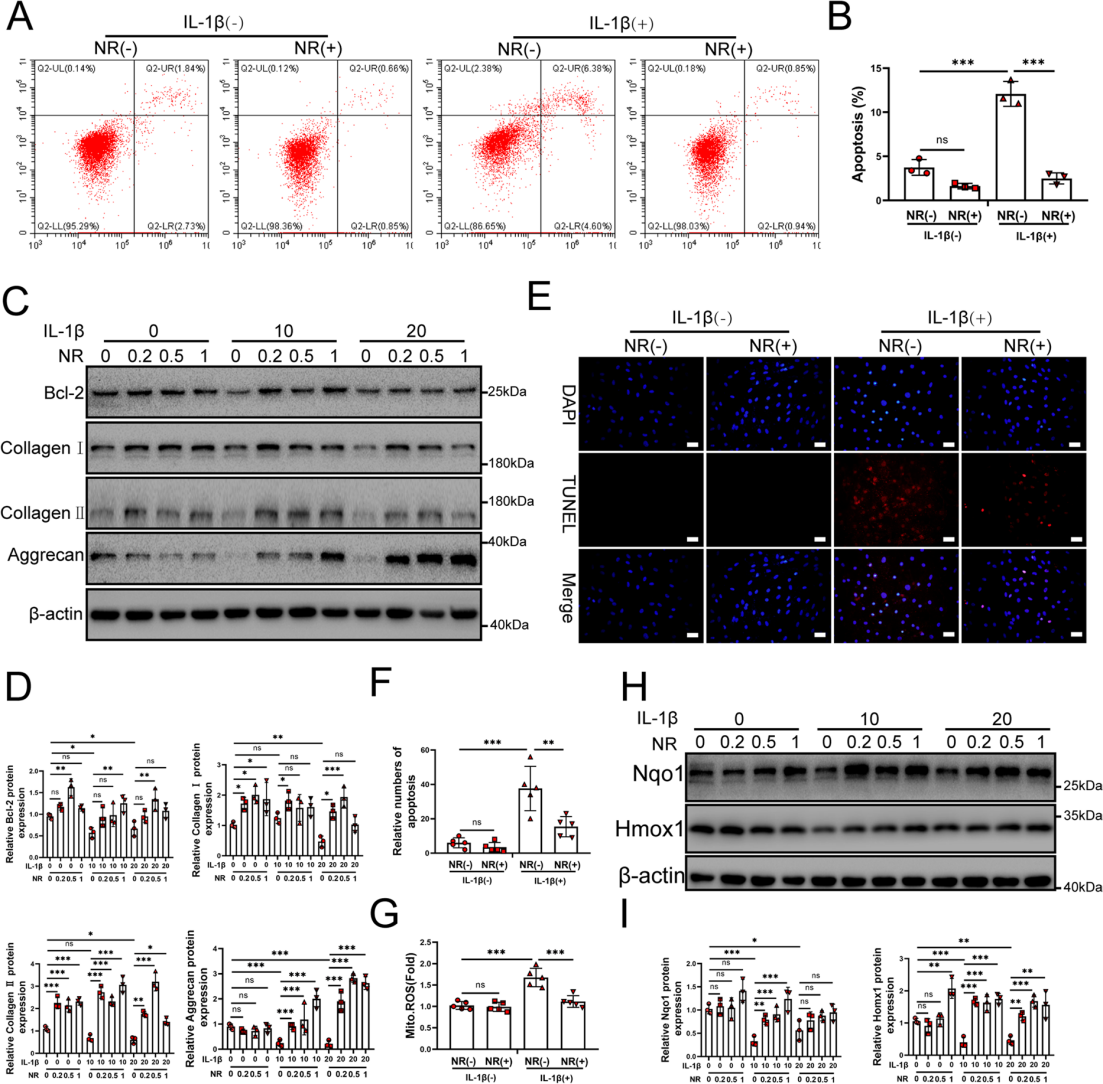
NR protected NP cells from IL-1β. (**A**) Flow cytometry to NP cell apoptosis (*n* = 3). NP cells were divided into 2 groups and treated with 0 ng/ml and 20 ng/ml IL-1β for 48 h respectively. Each group was subdivided into 2 groups and treated with 0 and 1 mM NR for 6 h respectively after IL-1β treatments. (**B**) The quantitative analysis to the results of Flow cytometry. (**C**) Western blot to Bcl-2, Collagen I, Collagen II, Aggrecan and β-actin in NP cells. NP cells were divided into 3 groups and treated with 0 ng/ml, 10 ng/ml and 20 ng/ml IL-1β for 48 h respectively (*n* = 3). Each group was subdivided into 4 groups and treated with 0, 0.2, 0.5 and 1 mM NR for 6 h respectively after IL-1β treatments. (**D**) The quantitative analysis to the results of Western blot. (**E**) TUNEL assays to NP cell apoptosis (*n* = 3). NP cells were divided into 2 groups and treated with 0 ng/ml and 20 ng/ml IL-1β for 48 h respectively. Each group was subdivided into 2 groups and treated with 0 and 1 mM NR for 6 h respectively after IL-1β treatments. The experimental grouping settings were indicated. Scale bars = 50 μm. (**F**) The quantitative analysis to the results of TUNEL assays. (**G**) Mitochondrial ROS were detected in NP cells. (**H**) Western blot to Nqo1, Homx1 and β-actin in NP cells. NP cells were divided into 3 groups and treated with 0 ng/ml, 10 ng/ml and 20 ng/ml IL-1β for 48 h respectively (*n* = 3). Each group was subdivided into 4 groups and treated with 0, 0.2, 0.5 and 1 mM NR for 6 h respectively after IL-1β treatments. (**I**) The quantitative analysis to the results of Western blot. Statistical significance was analyzed by one-way ANOVA followed by a post hoc Tukey’s test. All data were presented as mean ± SD. *p < 0.05; **p < 0.01; ***p < 0.001

To further confirm whether an increasing UPR^mt^ could effectively attenuate NP cell apoptosis, we performed flow cytometry assays to evaluate the level of apoptosis. NR treatment significantly reduced the apoptosis of NP cells induced by IL-1β (Fig. 4A and B). Degeneration in intervertebral discs has been associated with NP cell apoptosis (Xu et al. 2019a, b; Xie et al. 2021). Western blot results showed that Bcl-2 and extracellular matrix-related protein expression was promoted by NR (Fig. 4C and D). TUNEL assays results confirmed that NR could attenuate apoptosis (Fig. 4E and F). NR was found to reduce the production of mitochondrial reactive oxygen species (ROS) (Fig. 4G). NR also promoted the protein expression of the antioxidant genes Nqo1 and Homx1, indicating that the antioxidant capacity of NP cells was enhanced (Fig. 4H and I). These results confirmed that NR protected NP cells from apoptosis, improved mitochondrial function and enhanced matrix synthesis in NP cells.

### The UPR^mt^ induces mitophagy to protect NP cells from apoptosis

**Fig. 5:**
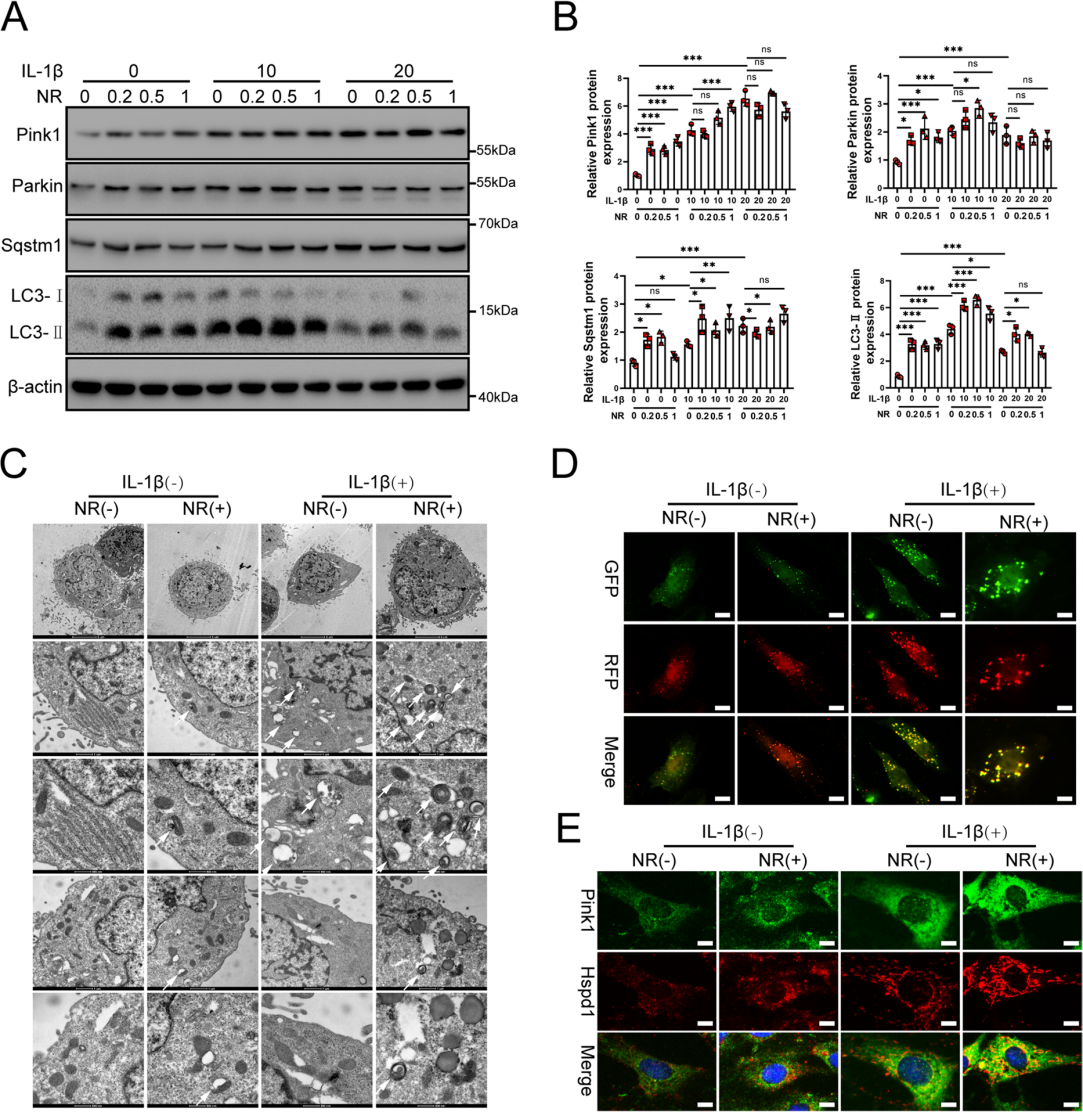
IL-1β caused mitophagy in NP cells. (**A**) Western blot to mitophagy markers including Pink1, Parkin, Sqstm1, LC3 and β-actin in NP cells. NP cells were divided into 3 groups and treated with 0 ng/ml, 10 ng/ml and 20 ng/ml IL-1β for 48 h respectively. Each group was subdivided into 4 groups and treated with 0, 0.2, 0.5 and 1 mM NR for 6 h respectively after IL-1β treatments. (**B**) The quantitative analysis to the results of Western blot. (**C**) Electron microscope was used to detect mitochondrial autophagosome in NP cells. Mitochondrial autophagosomes were indicated by white arrows. (**D**) NP cells stably expressing the stubRFP-sensGFP-LC3 fusion protein were established and observed by the fluorescence microscope (*n* = 3). sensGFP is sensitive to the pH changes owing to the fusion of autophagosomes and lysosomes, whereas mRFP is stable. When autophagy was induced, autophagosomes and lysosomes were fused, sensGFP was quenched and mRFP was increased. Scale bars = 5 μm. (**E**) Immunofluorescence analysis to mitophagy marker Pink1 and the UPR^mt^ marker Hspd1 in NP cells (*n* = 3). Scale bars = 5 μm.Statistical significance was analyzed by one-way ANOVA followed by a post hoc Tukey’s test. All data were presented as mean ± SD. *p < 0.05; **p < 0.01; ***p < 0.001

Mitochondrial unfolded proteins induce mitophagy by changing gene transcription and cellular activity (Pellegrino and Haynes 2015; Beck et al. 2015). However, whether the UPR^mt^ regulates mitophagy remains unclear. First, mitophagy was found to be induced by IL-1β. NR was evaluated to determine whether it could promote mitophagy (Fig. S3A-D). Mitophagy markers such as LC3-II, Sqstm1, Parkin and Pink1 were increased by IL-1β treatment of NP cells. As was shown in Fig. 5A and B, several important markers of mitophagy, such as LC3-II, Sqstm1, Parkin and Pink1, were further upregulated in NP cells after NR treatment.. Electron microscopy results showed that IL-1β increased mitophagy, while NR further enhanced mitophagy in NP cells (Fig. 5C). Mitochondrial autophagosomes were indicated by white arrows in Fig. 5C. Autophagosomes encapsulated damaged mitochondria and formed mitochondrial autophagosomes. Mitochondrial autophagosome and lysosome fused to form mature mitochondrial SKN-1. We observed the enhanced autophagosomal-lysosomal fusion process in NP cells using a fluorescence microscope (Fig. 5D). Immunofluorescence results showed that Pink1 was increased by NR (Fig. 5E). These results confirmed that the UPR^mt^ could induce mitophagy in NP cells.

### Upregulation of Atf5 induces UPR^mt^ to protect NP cells from IL-1β treatments

**Fig. 6:**
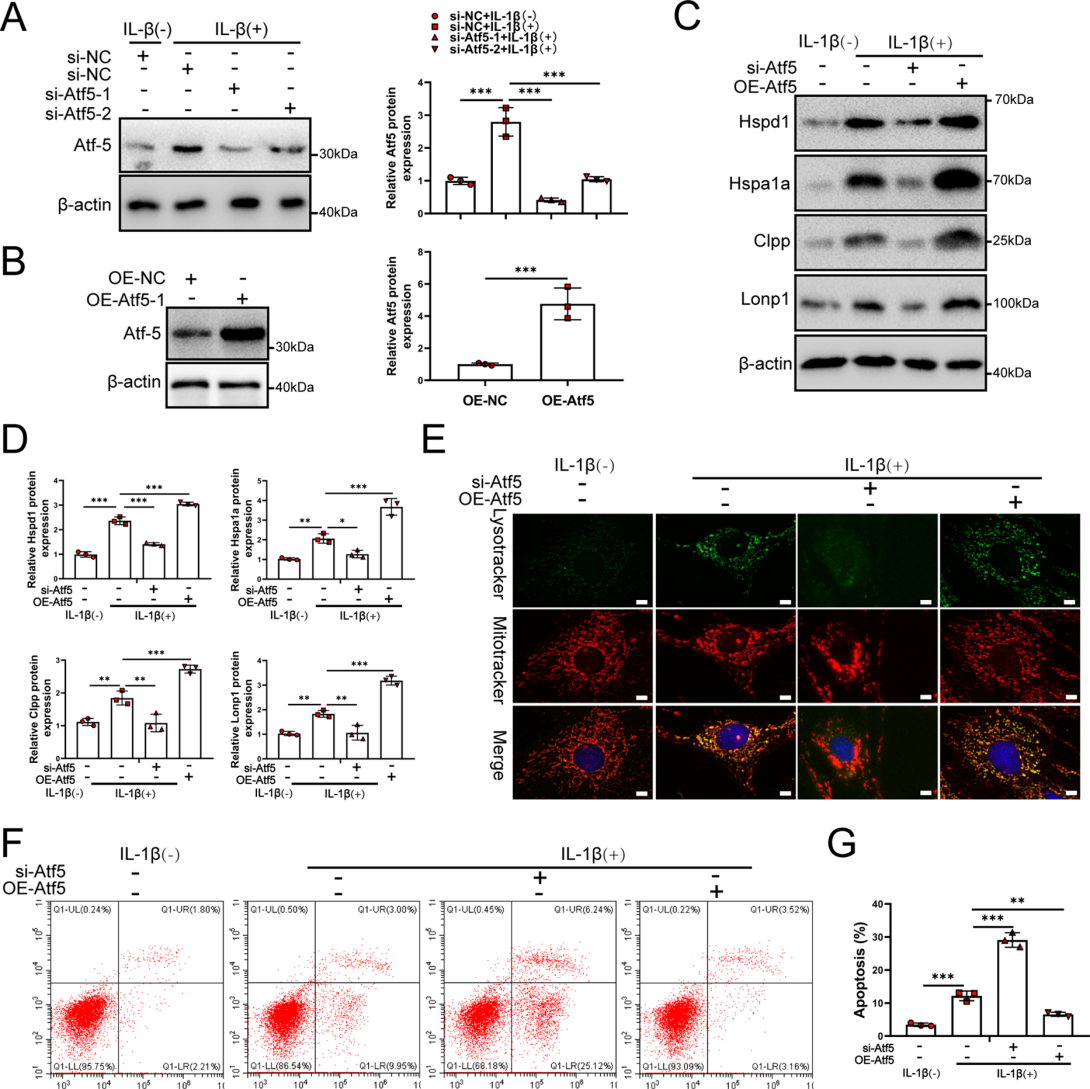
Atf5 mediated UPR^mt^ process to regulate NP cells. (**A**) NP cells were transfected with si-NC, si-Atf5-1 and si- Atf5-2 respectively (*n* = 3). NP cells were treated with 0 or 20 ng/ml IL-1β treatment for 48 h. The experimental grouping settings were indicated. (**B**) NP cells were transfected with OE-NC, OE-Atf5 (*n* = 3). (**C**) Western blot to the UPR^mt^ markers such as Hspd1, Hspa1a, Clpp, Lonp1, and β-actin in NP cells (*n* = 3). The experimental grouping settings were indicated. (**D**) The quantitative analysis to the results of Western blot. (**E**) Lysotracker and mitotracker staining were performed to assess the delivery of mitochondria to lysosome. The experimental grouping settings were indicated. (**F**) Flow cytometry to NP cell apoptosis (*n* = 3). (**G**) The quantitative analysis to the results of Flow cytometry. The experimental grouping settings were indicated. Scale bars = 5 μm. Statistical significance was analyzed by one-way ANOVA followed by a post hoc Tukey’s test. All data were presented as mean ± SD. *p < 0.05; **p < 0.01; ***p < 0.001

Previous studies reported that Atf5 could enhance UPR^mt^ (Zhou et al. 2022; Gao et al. 2021; Smyrnias et al. 2019). To investigate whether Atf5 induces UPR^mt^ to protect NP cells, small interfering RNAs for Atf5 (si-Atf5) were designed to silence Atf5 expression and Atf5 overexpression plasmid were used to upregulate Atf5 expression in NP cells (Fig. 6A and B). Atf5 knockdown could inhibit UPR^mt^ while upregulation of Atf5 increased UPR^mt^ in NP cells (Fig. 6C and D). Moreover, upregulation of Atf5 could reduce the apoptosis induced by IL-1β while silencing of Atf5 increased the effect of IL-1β (Fig. 6F and G). Upregulation of Atf5 increased the fusion of mitochondria and lysosome. Silencing of Atf5 reduced the fusion of mitochondria and lysosome (Fig. 6E). These results confirmed that Atf5 induced UPR^mt^ to cooperating with mitophagy, thereby protected NP cells from IL-1β treatments.

### Silencing of Pink1 reverses the protective effects of NR and inhibits mitophagy induced by the UPR^mt^

**Fig. 7:**
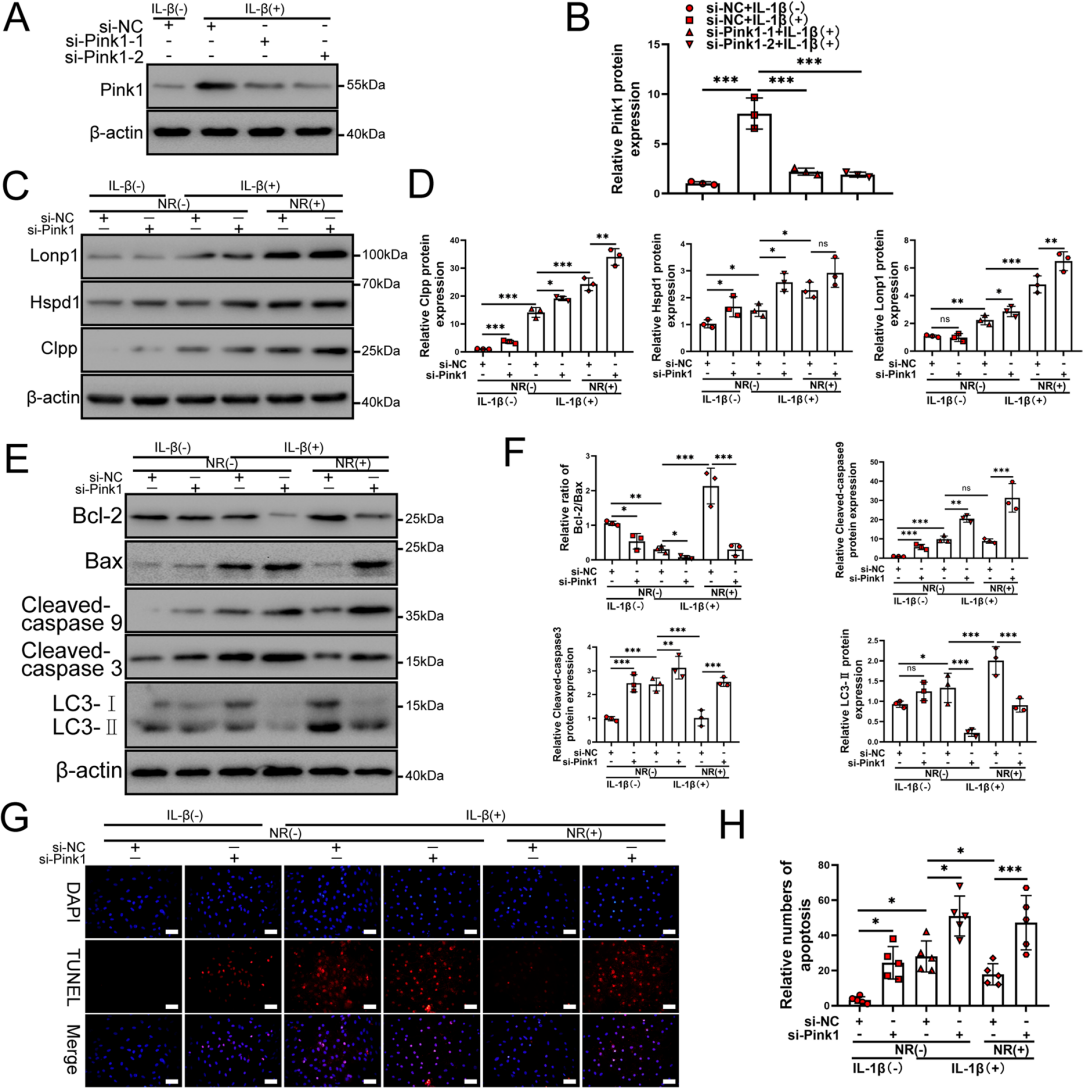
Silencing of Pink1 reduced the protective effect of NR on NP cells. (**A**) NP cells were transfected with si-NC, si-Pink1-1 and si-Pink1-2 respectively (*n* = 3). NP cells were treated with 0 or 20 ng/ml IL-1β treatment for 48 h. The experimental grouping settings were indicated. (**B**) The quantitative analysis to the results of Western blot. (**C**) Western blot to the UPR^mt^ markers including Lonp1, Hspd1, Clpp and β-actin in NP cells (*n* = 3). The experimental grouping settings were indicated. (**D**) The quantitative analysis to the results of Western blot. (**E**) Western blot to Bcl-2, Bax, Cleaved-caspase 3, Cleaved-caspase 9, LC3-II and β-actin in NP cells (*n* = 3). (**F**) The quantitative analysis to the results of Western blot. (**G**) TUNEL assays to NP cell apoptosis (*n* = 3). The experimental grouping settings were indicated. NP cells were transfected with si-NC or si-Pink1, treated with 0 ng/ml and 20 ng/ml IL-1β for 48 h and then treated with 0 and 1 mM NR for 6 h. The experimental grouping settings were indicated. Scale bars = 50 μm. (**H**) The quantitative analysis to the results of TUNEL assays. Statistical significance was analyzed by one-way ANOVA followed by a post hoc Tukey’s test. All data were presented as mean ± SD. *p < 0.05; **p < 0.01; ***p < 0.001

To further investigate whether the UPR^mt^ induces mitophagy to protect NP cells, we constructed small interfering RNA (si-Pink1) for Pink1 and negative control small interfering RNA (si-NC) to silence the expression of Pink1, a known key mediator of mitophagy (Narendra et al. 2010). Western blot assays confirmed that Pink1 expression was reduced by si-Pink1-1 and si-Pink1-2 (Fig. 7A and B). Si-Pink1-2 was used in subsequent experiments. Silencing of Pink1 did not change the level of the UPR^mt^ (Fig. 7C and D), which showed that Pink1 was not upstream of the UPR^mt^. When Pink1 was knocked down by si-Pink1, NR did not enhance the level of mitophagy. Although NP cells were treated with NR, silencing Pink1 largely alleviated NR protection in NP cells after IL-1β treatment. Pink1 knockdown increased the apoptosis of NP cells exposed to IL-1β (Fig. 7E and F). The results of TUNEL assays confirmed the above findings (Fig. 7G and H). These results confirmed that the UPR^mt^ induced mitophagy to protect NP cells.

### NR attenuates IVDD in rats via the UPR^mt^

**Fig. 8:**
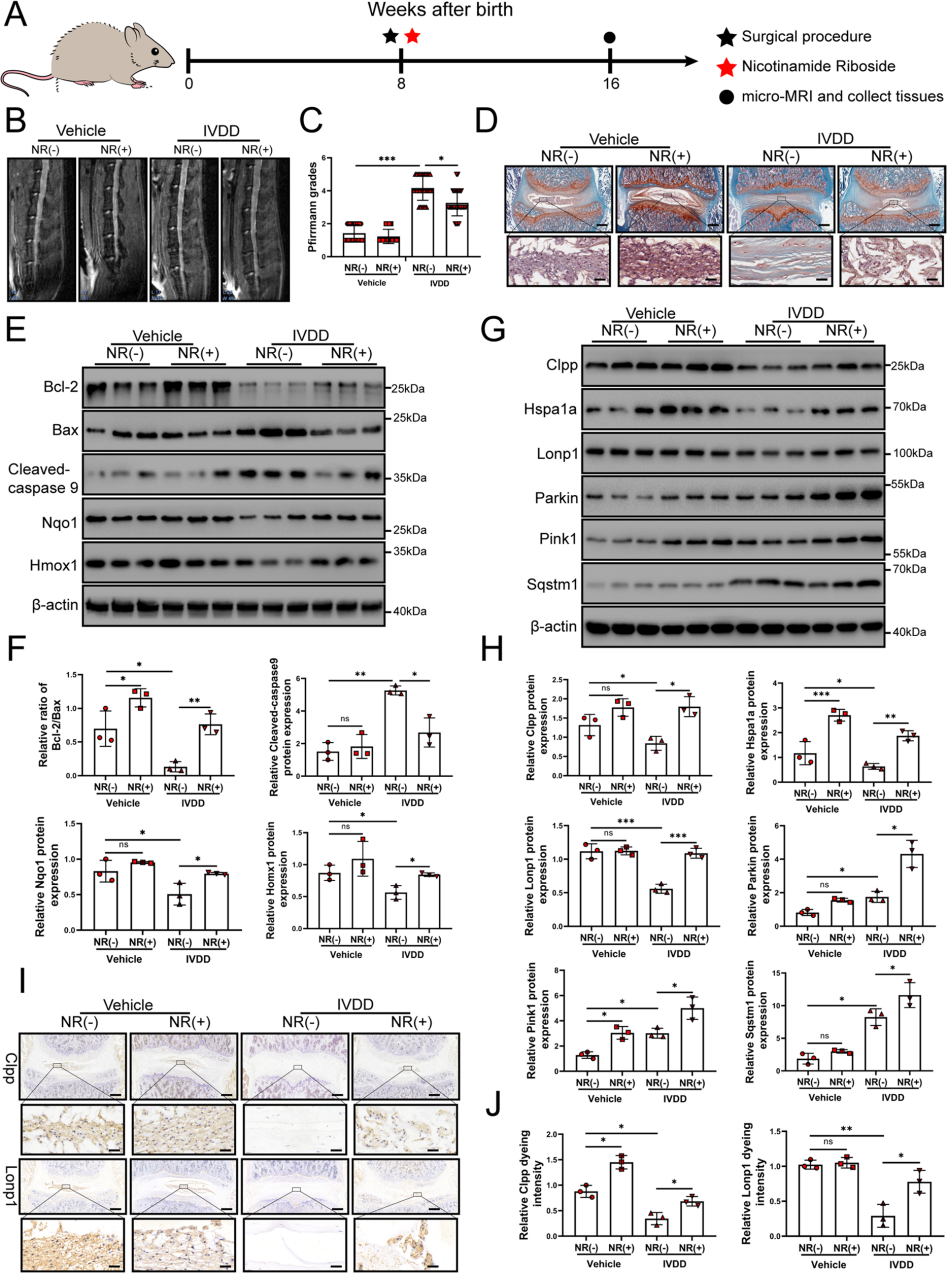
NR might attenuate the IVDD of rat. Rats were divided randomly into sham operation group and unbalanced dynamic group. Sham operation groups were subdivided into NR (-) and NR ( +) (400 mg/kg/day) group. Per group included six rats. (**A**) Schematic diagram of animal experimental schedule. (**B**) T2-weighted MRI of IVDD models from each group at 16 weeks (*n* = 6). (**C**) Pfirrmann MRI scores for T2-weighted MRIs of IVDD models from each group at 4 months (*n* = 30). Each rat including L1-2, L2-3, L3-4, L4-5 and L5-6 were evaluated by Pfirrmann MRI scores according to T2-weighted MRIs (*n* = 30). Each rat had a total of five segments of intervertebral discs evaluated by Pfirrmann MRI scores according to T2-weighted MRIs. There were six rats in each group, with a total of 30 segments intervertebral discs. (**D**) Safranin O-fast green staining showed the structure of the intervertebral disc (*n* = 6). Scale bars = 200 μm and 2.5 μm. (**E**) Western blot to Bcl-2, Bax, Cleaved-caspase 9, Nqo1, Homx1 and β-actin in the tissue samples of rat IVDD (*n* = 3). (**F**) The quantitative analysis to the results of Western blot. (**G**) Western blot to the UPR^mt^ markers such as Clpp, Hspa1a, Lonp1 and mitophagy markers such as Pink1, Parkin, sqstm1 and β-actin in the tissue samples of rat IVDD (*n* = 3). (**H**) The quantitative analysis to the results of Western blot. (**I**) Immunohistochemical examination of the UPR^mt^ markers including Clpp and Lonp1 in the tissue samples of rat IVDD (*n* = 6). Scale bars = 200 μm and 2.5 μm. (**J**) The quantitative analysis to the results of Immunohistochemistry.Statistical significance was analyzed by one-way ANOVA followed by a post hoc Tukey’s test. All data were presented as mean ± SD. *p < 0.05; **p < 0.01; ***p < 0.001

In vivo, NR was able to alleviate IVDD in rats. A schematic diagram of the animal experimental schedule was shown in Fig. 8A. Micro-MRI results showed that the Pfirrmann score of intervertebral discs was lower after NR treatment in rats with surgery (Fig. 8B and C). Lower grades were mainly observed in L1-2, L2-3, L3-4, L4-5 and L5-6 in the rats with IVDD according to level-by-level analysis of disc degeneration scores. Histological analysis of lumbar discs demonstrated that NR could significantly further relieve the damaged disc structure of rats and better preserve the tissue and cell morphology than those of the vehicle-treated animals. There were very few NP cells in the degenerative disc. However, we saw quite a few NP cells in the degenerative intervertebral disc of the NR-treated group (Fig. 8D and Fig. S4A). To verify whether the protective effect on the discs was due to the reduction in apoptosis and increase in oxidation resistance, we detected the apoptotic markers and antioxidative genes of discs from rats. Intervertebral discs from the rats treated with NR showed a reduced Bax/Bcl-2 ratio and cleaved-caspase 9 as well as increased levels of Nqo-1 and Homx-1, suggesting that NR could reduce apoptosis and increase antioxidant function in intervertebral discs (Fig. 8E and F). Furthermore, senescence markers including p21 and p53 protein were increased in degenerative intervertebral discs of rats while they were reduced after NR treatment (Fig. S4B). Therefore, these results demonstrated that NR played a protective role in IVDD in rats.

To investigate whether the UPR^mt^ is involved in this process, we evaluated the level of the UPR^mt^ in the intervertebral discs. Western blot results confirmed that the Clpp and Lonp1 was increased in IVDD of rats after NR treatments (Fig. 8G and H). The immunohistochemistry results showed that Clpp and Lonp1 were increased in IVDD tissue of rats after NR treatment (Fig. 8I and J). NR treatments of the rats with IVDD increased the expression of mitophagy markers (Fig. 8G and H). Interestingly, increased Parkin and Pink1 expression was observed in the tissue of the rats with IVDD. Higher levels of Parkin and Pink1 appeared in the IVDD + NR compared to the IVDD group. This finding was also observed in the tissue of human IVDD. During early IVDD, there are other mechanisms that activate mitophagy, and promotion of the UPR^mt^ further activates mitophagy. These results indicated that UPR^mt^ was involved in IVDD through mitophagy.

## Discussion

Better study of the pathogenesis of IVDD is great significance for the effective treatment of low back pain. NP cell apoptosis is a key factor of IVDD. The response in which mitochondrial dysfunction leads to the accumulation of large amounts of unfolded proteins and induces changes in gene transcription and cell cytoactive is called UPR^mt^ (Shpilka and Haynes 2017; Jovaisaite and Auwerx 2014). Mitochondrial dysfunction leads to accumulation of large numbers of unfolded proteins in the mitochondria, which induces nuclear encoding mitochondrial chaperone proteins and proteases, such as HSPA1A, HSPA9, HSPE1, YME1L1, CLPP, LONP1 and other proteins, which are upregulated to improve mitochondrial function (Zhang et al. 2018; Melber and Haynes 2018) and delaying aging (Zhang et al. 2016). During mitochondrial stress, metabolic genes is altered by UPR^mt^ (Nargund et al. 2012). Similar to the increased UPR^mt^, mitophagy regulated by PINK1 and Parkin, as well as SKN-1, also prolongs the lifespan (Palikaras et al. 2015). SKN-1 is composed of ATFS-1 and other mitochondrial autophagy components during mitochondrial stress (Nargund et al. 2012). UPR^mt^ and mitophagy can be activated simultaneously when severely damaged mitochondria were cleared because both respond to mitochondrial dysfunction (Shpilka and Haynes 2017). The respective responsibilities of UPR^mt^ and mitophagy are fairly clear. UPR^mt^ promotes transcriptional adaptation for mitochondrial function. Mitophagy promotes the degradation of severely damaged mitochondria (Shpilka and Haynes 2017). However, how they will be coordinated to restore the mitochondrial network remains unclear. Herein, our study demonstrated that Atf5 caused by IL-1β could induce UPR^mt^ to delay the process of apoptosis-induced IVDD through mitophagy (Feng et al. 2017). When mitophagy was inhibited, the UPR^mt^ was less effective at protecting NP cells.

We found that the level of the UPR^mt^ in human IVDD tissue was obviously decreased with the increased degree of IVDD. Western blot experiments further confirmed the results of our findings. However, the results of Immunohistochemical examination showed that HSPA1A and HSPD1 protein expression was increased in human IVDD tissues. Hu et al. (Hu et al. 2022) also confirmed the results of Immunohistochemical examination. There were more NP cells in disc tissue than in degenerative disc tissue (Figure S1). To date, the loss of NP cells is a major driver of IVDD (Risbud and Shapiro 2013; Wang et al. 2015). NP cells in the degenerative disc tissue were decreased and hypertrophic chondrocyte-like differentiation of resident cells were increased. What’s more, there were many other cells such as macrophages, neutrophils, annulus fibrosus cell and endothelial cells in the degenerative disc tissue (Risbud and Shapiro 2013). Therefore, the level of UPR^mt^ was upregulated in NP cells but not in other cells at this time. These results explained why the expression of UPR^mt^ markers was elevated in the in vitro model of IVDD but reduced in human tissue specimens according to the results of Western blot.

IL-1β is involved in the process of IVDD (Jia et al. 2019). IL-1β is a key mediator of mechanical load and chronic inflammation inducing IVDD (Walter et al. 2011). IL-1β is widely used to simulate NP cells in vitro (Chen et al. 2020; Dong et al. 2019). Therefore, IL-1β was used to treat NP cells. After treating NP cells, we found that the mitochondrial function of NP cells was significantly weaken. Then, the level of the UPR^mt^ was increased prominently in NP cells. The results in human intervertebral disc tissues might be contrary to the in vitro results, which indicated that disorder of the UPR^mt^ was an important cause of IVDD. IL-1β activated the UPR^mt^ in NP cells in the early stage of IVDD. The activated UPR^mt^ protects NP cells and inhibits IL-1β destruction. In the long degeneration process, the UPR^mt^ was gradually destroyed.

To prove our hypothesis, UPR^mt^ inducer NR was used to further activate the UPR^mt^ in NP cells. NR could further induce the level of the UPR^mt^ in NP cells. The results confirmed our hypothesis that NR could protect the mitochondrial function of NP cells. IL-1β caused increased level of ATP in NP cells. This finding might be related to the decrease in the level of intercellular matrix synthesis by NP cells. The decreased matrix synthesis reduced the consumption of ATP and increased the level of ATP in NP cells. NR significantly inhibited NP cell apoptosis and promoted Collagen I, Collagen II and Aggrecan expression in NP cells. NR could also promote the expression of antioxidant genes. These findings indicated that the UPR^mt^ was able to protect NP cells from IL-1β disruption. In the process of IVDD, the inhibition of the UPR^mt^ is an important factor.

Autophagy can eliminate damaged and useless organelles to avoid apoptosis (Ciechomska 2019; Denton and Kumar 2018). Oxidative stress induces autophagy, which eliminates damaged organelles (such as mitochondria) and aggregated proteins (Ureshino et al. 2014). IL-1β exposure induces many oxidative reactions (Chen et al. 2017). How moderate mitophagy induces protection in NP cells is still unknown. Our study confirmed the results of previous studies that IL-1βpromoted autophagy and mitophagy of NP cells. Interestingly, NR was capable of further increasing mitophagy level in NP cells. Importantly, silencing Pink1 impaired the protective role of NR in NP cells. These results indicated that the UPR^mt^ protected NP cells by inducing mitophagy. The results in animals further confirmed our hypothesis that the UPR^mt^ agonist NR could alleviate IVDD in rats and simultaneously increase the levels of the UPR^mt^ and mitophagy in the intervertebral discs of rats.

There has been increasing evidence that excess ROS activates IVDD by regulating stromal metabolism, pro-inflammatory phenotype, autophagy, aging, and apoptosis of NP cells (Davalli et al. 2016; Kang et al. 2020; Cao et al. 2022). Each pathological process affects each other, for example, proper autophagy can alleviate NP cell senescence and apoptosis. Apoptosis is one of the outcomes of cellular senescence. The main mode of NP cell death is apoptosis, and the rest of the death modes such as ferroptosis and pyroptosis are also available. Several studies have reported a cellular senescence phenotype of degenerative intervertebral discs in humans and have shown a correlation between cellular senescence and disc degeneration (Roberts et al. 2006; Gruber et al. 2007a; Maitre et al. 2007). In addition, it has been shown that the number of senescent disc cells increases as disc degeneration progresses (Maitre et al. 2007; Gruber et al. 2007b). Although the relationship between aging and degeneration in IVDD is very complex, the models that study aging-related IVDD are different from those that predominantly degeneration. According to previous studies (Han et al. 2020; Novais et al. 2020; Mohanty et al. 2019; Patil et al. 2019), age-related IVDD models are mainly based on older animals such as 2-year-old mice or rats, as well as genetically engineered aging animals. These models do not involve surgical procedures. In vivo experiments of our study, the model was an 8-week-old healthy male rat in the youth stage. Rats had a surgery in IVDD group. There were other surgical procedures (Jia et al. 2024; Wang et al. 2006; Shi et al. 2024; Zhang et al. 2024). p21 and p53 protein expression were increased in degenerative discs of rats while they were reduced after NR treatment. These results showed that UPR^mt^ could attenuate senescence and degeneration of intervertebral discs. Therefore, the observed differences correlated with both age and degeneration, suggesting that the surgery associated IVDD model also underwent senescence.

This study identifies the following issues for further study. First, the role of the UPR^mt^ marker genes including Clpp, Hspd1, and Lonp1needs to be confirmed in IVDD. Second, our study demonstrated that Atf5 caused by IL-1β could induce UPR^mt^ to delay the process of IVDD through mitophagy. However, there were many UPR^mt^ markers and it was difficult to determine whether UPR^mt^ markers acted directly on target genes of mitophagy to regulate mitophagy. The underlying mechanism by which activated mitophagy remains unclear in this study. Therefore, the underlying connection between UPR^mt^ and mitophagy need further investigation in the future. Thirdly, for the human study, number of control samples was low. Further follow-up experiments were needed after control sample collection was sufficient.

**Fig. 9:**
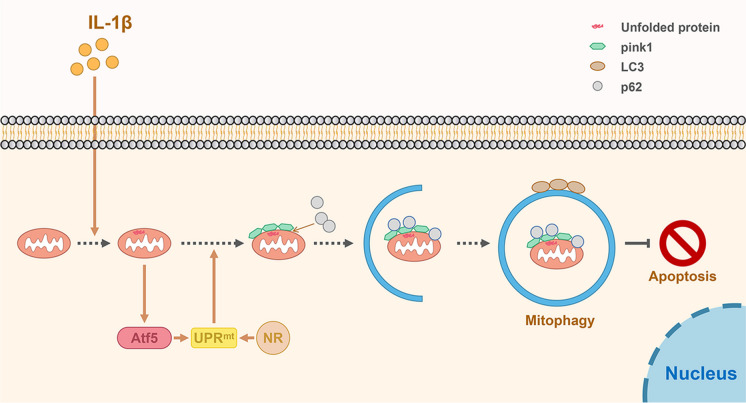
A schematic diagram for the underlying mechanism of the UPR^mt^ in regulating IVDD. Mitochondrial unfold proteins caused by IL-1β stimulate Atf5 to induce UPR^mt^, thereby promoting mitophagy to protect NP cell from apoptosis

In conclusion, the UPR^mt^ agonist NR induced mitophagy to inhibit apoptosis and ameliorate the metabolism of NP cells and then alleviated disc degeneration (Fig. 9). Our study confirmed that in the normal intervertebral disc, UPR^mt^ activated mitophagy to remove dysfunctional mitochondria and avoided the apoptosis of NP cells triggered by dysfunctional mitochondria addition to clearing unfolded or misfolded proteins of mitochondria.

## Supplementary Information

Below is the link to the electronic supplementary material.

Supplementary file1 (TIF 1886 KB)

Supplementary file2 (TIF 469 KB)

Supplementary file3 (TIF 633 KB)

Supplementary file4 (TIF 5361 KB)

Supplementary file5 (DOCX 38 KB)

Supplementary file6 (DOCX 20 KB)

Supplementary file7 (DOCX 25 KB)

Supplementary file8 (DOCX 28 KB)

## Data Availability

The datasets generated during and/or analyzed during the current study are available from the corresponding author on reasonable request.
