# Supplementary figures and images for: The mitochondrial UPR induced by ATF5 attenuates intervertebral disc degeneration via cooperating with mitophagy
Source: Cell Biol Toxicol. 2024 Mar 13;40(1):16. doi: 10.1007/s10565-024-09854-9 (PMC10933207; doi:10.1007/s10565-024-09854-9)

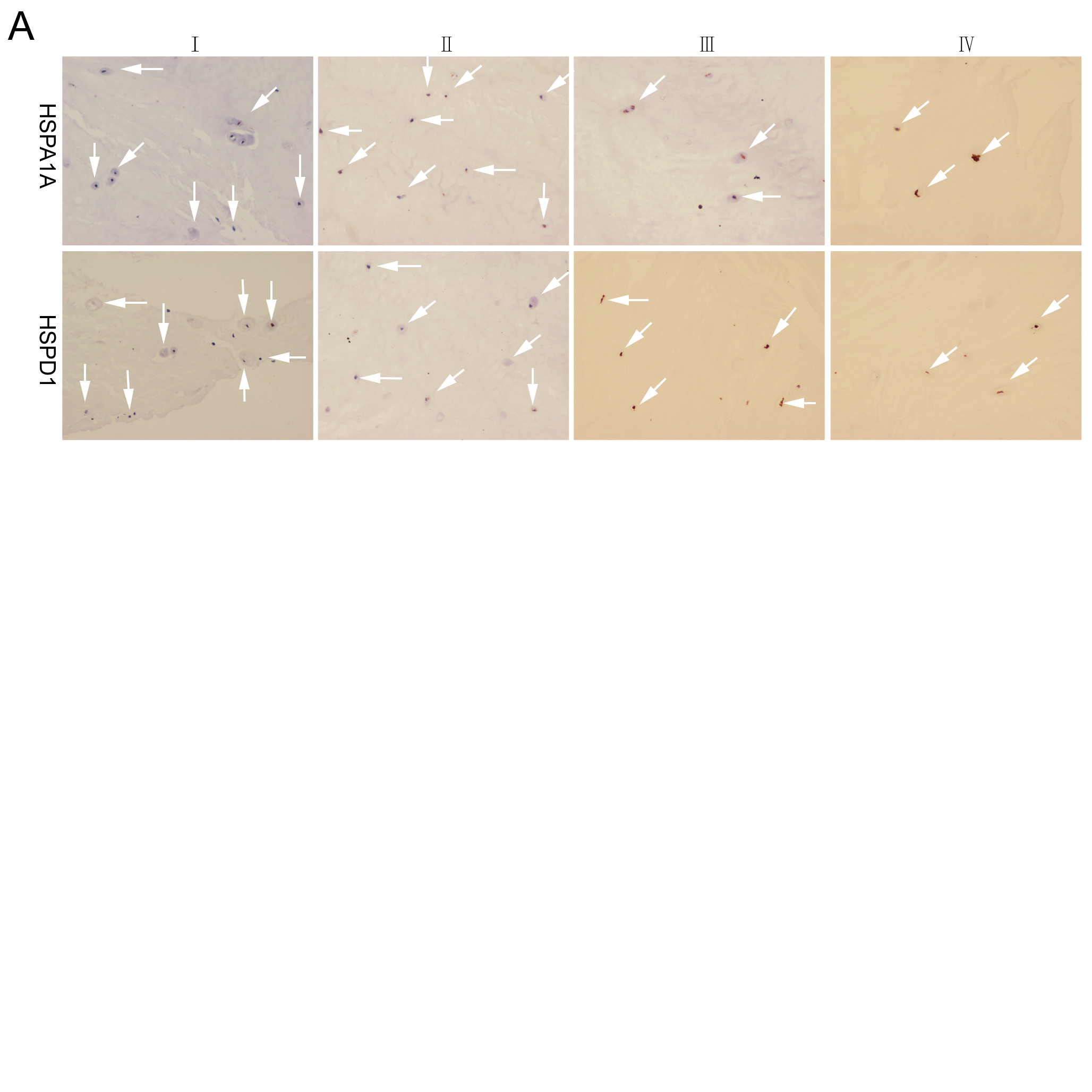

Supplement: Supplementary file 1 — Supplementary file1 (TIF 1886 KB) [file 10565_2024_9854_MOESM1_ESM.tif]

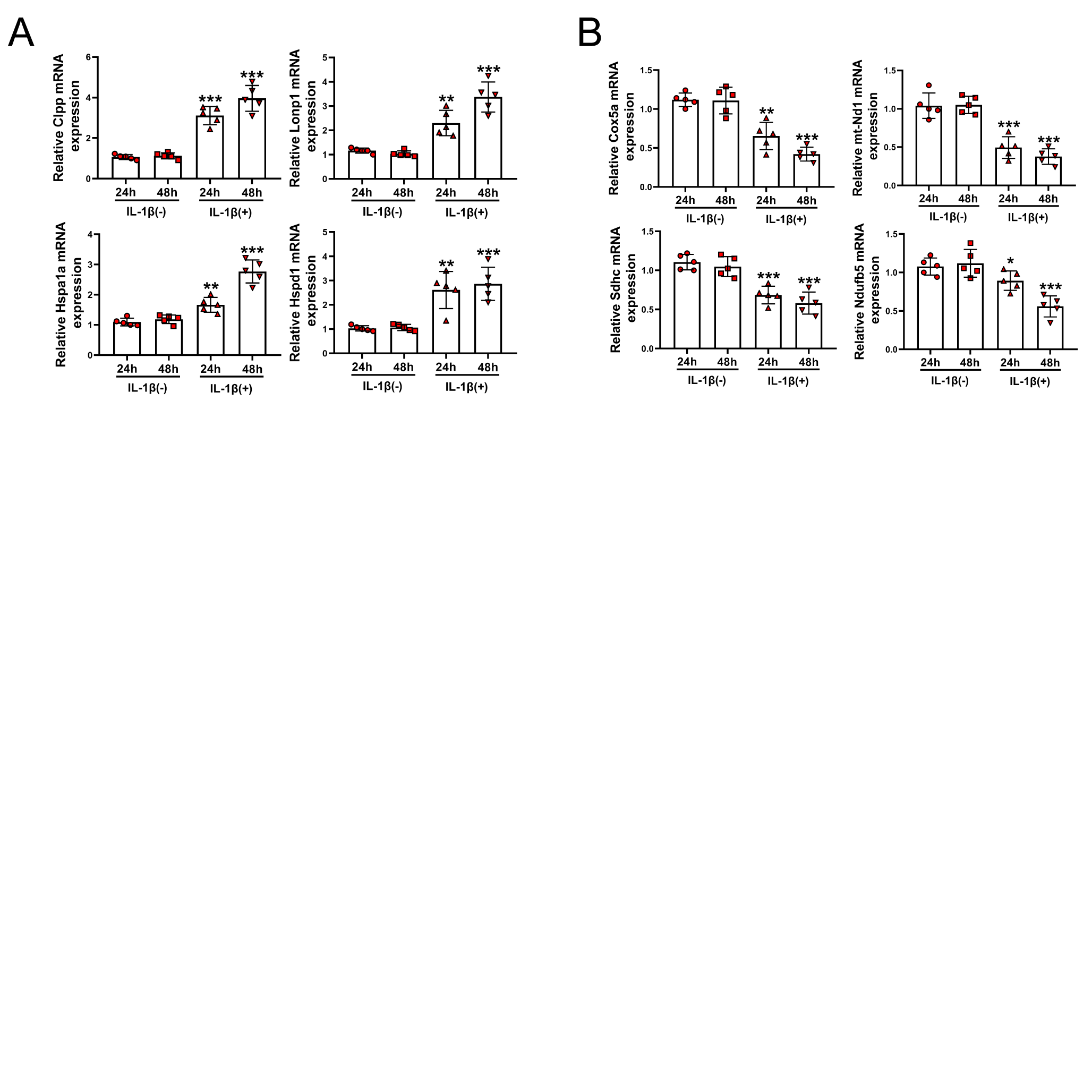

Supplement: Supplementary file 2 — Supplementary file2 (TIF 469 KB) [file 10565_2024_9854_MOESM2_ESM.tif]

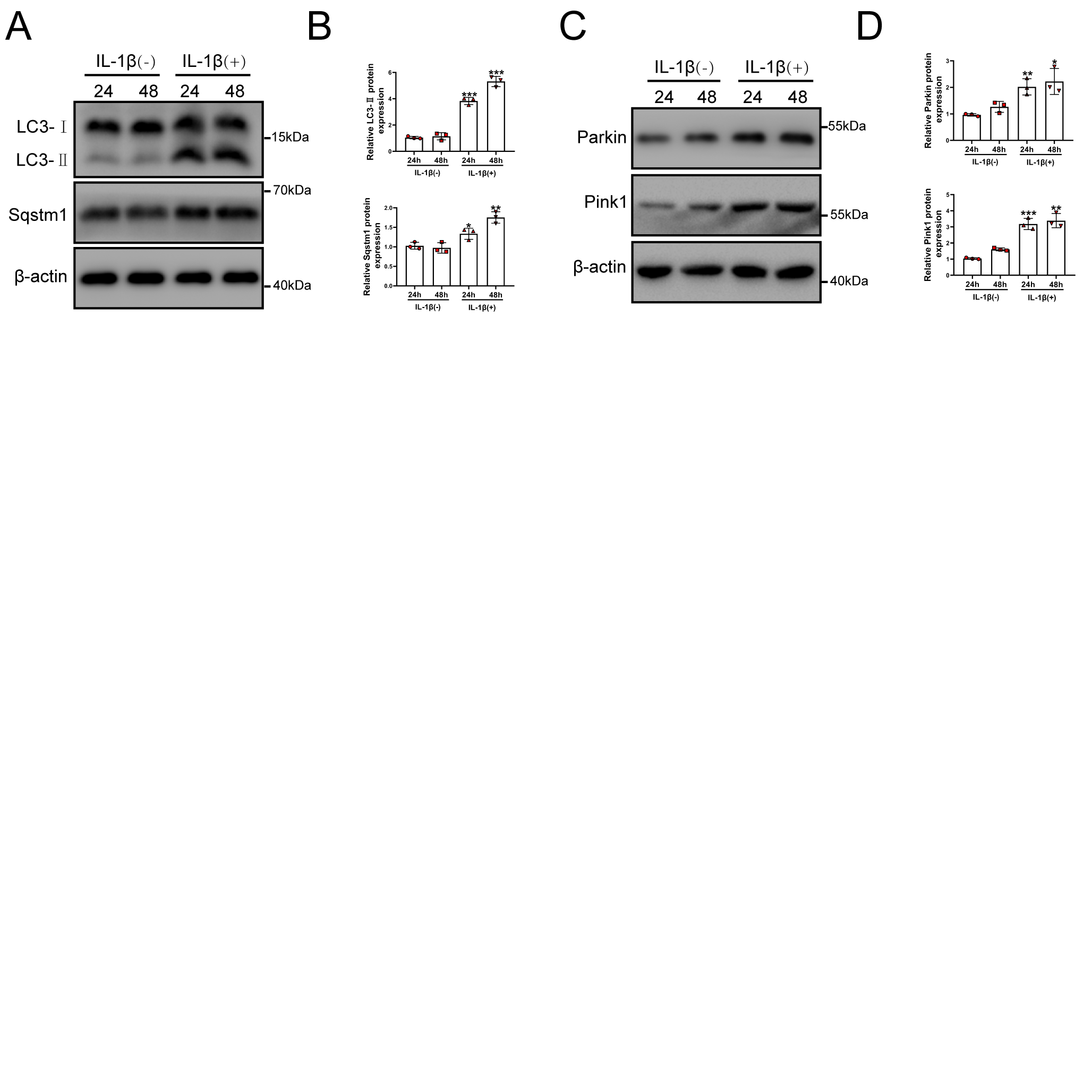

Supplement: Supplementary file 3 — Supplementary file3 (TIF 633 KB) [file 10565_2024_9854_MOESM3_ESM.tif]

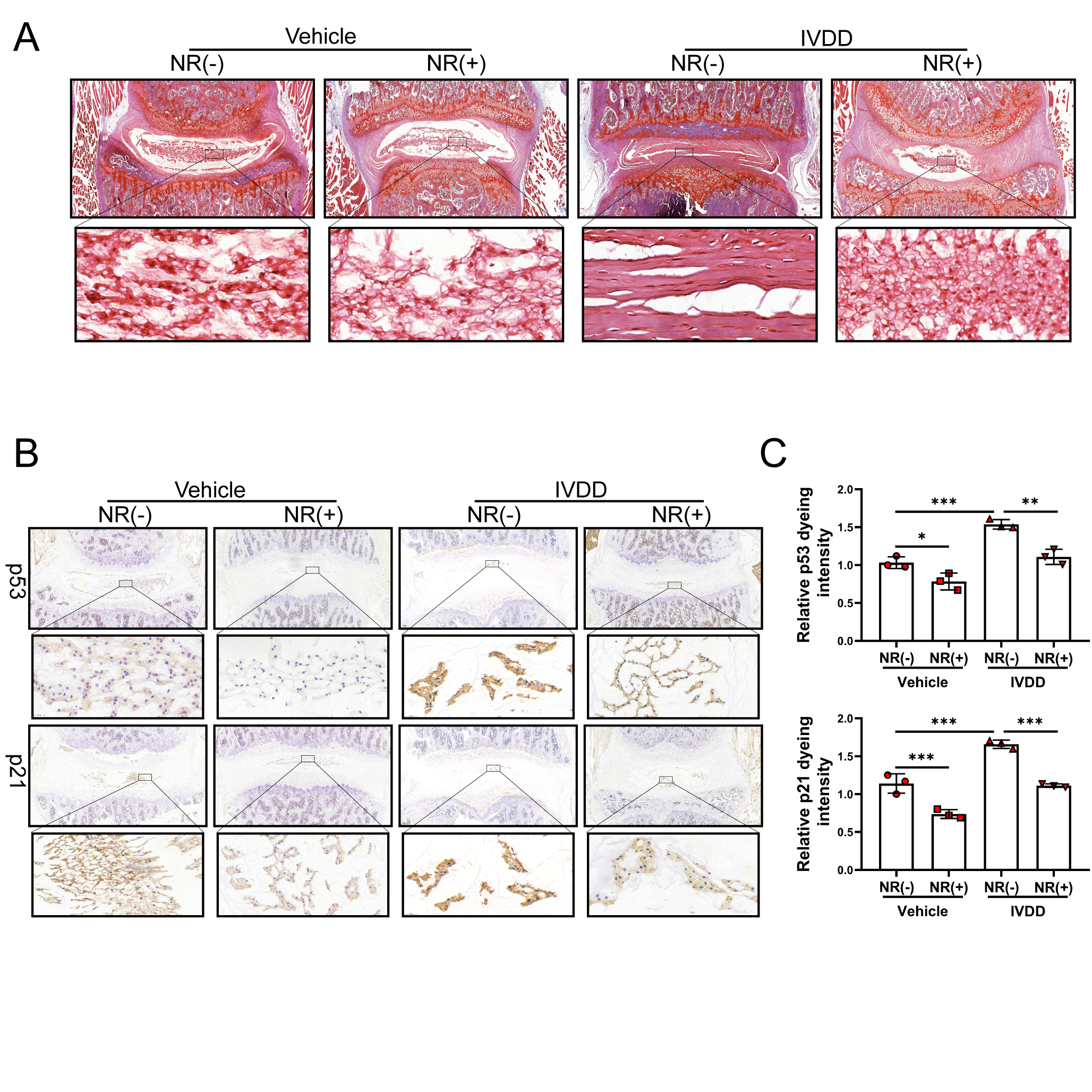

Supplement: Supplementary file 4 — Supplementary file4 (TIF 5361 KB) [file 10565_2024_9854_MOESM4_ESM.tif]
