# Supplementary material for: The mitochondrial UPR induced by ATF5 attenuates intervertebral disc degeneration via cooperating with mitophagy
Source: Cell Biol Toxicol. 2024 Mar 13;40(1):16. doi: 10.1007/s10565-024-09854-9 (PMC10933207; doi:10.1007/s10565-024-09854-9)
Supplement: Supplementary file 5 — Supplementary file5 (DOCX 38 KB) [file 10565_2024_9854_MOESM5_ESM.docx]

Table 1 Patient information

| NO. | Gender | Age | Pfirrmann grades | Surgery |
| --- | --- | --- | --- | --- |
| 1 | M | 11 | 1 | HR |
| 2 | M | 5 | 1 | HR |
| 3 | M | 12 | 1 | HR |
| 4 | F | 6 | 1 | HR |
| 5 | M | 37 | 2 | PELD |
| 6 | M | 61 | 2 | PELD |
| 7 | F | 49 | 2 | PELD |
| 8 | M | 41 | 2 | PELD |
| 9 | F | 47 | 2 | PELD |
| 10 | F | 46 | 2 | PELD |
| 11 | M | 51 | 2 | PELD |
| 12 | M | 42 | 2 | PELD |
| 13 | F | 39 | 2 | PELD |
| 14 | M | 41 | 2 | PELD |
| 15 | M | 45 | 2 | PELD |
| 16 | M | 51 | 3 | TLIF |
| 17 | F | 68 | 3 | TLIF |
| 18 | F | 49 | 3 | TLIF |
| 19 | M | 55 | 3 | PELD |
| 20 | F | 51 | 3 | PELD |
| 21 | M | 60 | 3 | TLIF |
| 22 | F | 65 | 3 | PELD |
| 23 | F | 55 | 3 | PELD |
| 24 | F | 61 | 3 | TLIF |
| 25 | M | 58 | 3 | PELD |
| 26 | F | 53 | 3 | TLIF |
| 27 | M | 58 | 4 | TLIF |
| 28 | F | 54 | 4 | TLIF |
| 29 | F | 68 | 4 | TLIF |
| 30 | F | 67 | 4 | TLIF |
| 31 | M | 73 | 4 | TLIF |
| 32 | M | 71 | 4 | TLIF |
| 33 | F | 66 | 4 | TLIF |
| 34 | F | 73 | 4 | TLIF |
| 35 | F | 76 | 5 | TLIF |
| 36 | F | 73 | 5 | TLIF |
| 37 | M | 64 | 5 | TLIF |
| 38 | M | 67 | 5 | TLIF |
| 39 | F | 58 | 5 | TLIF |

NO. (Number); M (male); F (female); HR (Hemivertebra Resection); PELD (Percutaneous Endoscopic Lumbar Discectomy); TLIF (Transforaminal Lumbar Interbody Fusion)
