# Supplementary material for: The mitochondrial UPR induced by ATF5 attenuates intervertebral disc degeneration via cooperating with mitophagy
Source: Cell Biol Toxicol. 2024 Mar 13;40(1):16. doi: 10.1007/s10565-024-09854-9 (PMC10933207; doi:10.1007/s10565-024-09854-9)
Supplement: Supplementary file 6 — Supplementary file6 (DOCX 20 KB) [file 10565_2024_9854_MOESM6_ESM.docx]

Table 2 The sequences of siRNA

| siRNA | siRNA sequence (5’-3’) |
| --- | --- |
| Si-Pink1-1 | CGCAAATGTGCTTCATCTA |
| Si-Pink1-2 | CCTATGAAATCTTCGGGCT |
| Si-Atf5-1 | AAGUCAGCUGCUCUCAGGUAC |
| Si-Atf5-2 | CCUGUCCCUCCAUUUCACUTT |
