# Supplementary material for: The mitochondrial UPR induced by ATF5 attenuates intervertebral disc degeneration via cooperating with mitophagy
Source: Cell Biol Toxicol. 2024 Mar 13;40(1):16. doi: 10.1007/s10565-024-09854-9 (PMC10933207; doi:10.1007/s10565-024-09854-9)
Supplement: Supplementary file 7 — Supplementary file7 (DOCX 25 KB) [file 10565_2024_9854_MOESM7_ESM.docx]

Table 3 The sequence of primers

| Gene | Forward (5’-3’) | Reverse (5’-3’) |
| --- | --- | --- |
| Hspd1 | CACCACCACTGCCACTGTTCTG | CAACAGCCAACATCACACCTCTCC |
| Yme1l1 | CAGTTCCAAGGCCAGATGTGAAGG | TGCTCCAGAAAACCCAACAGTTCC |
| Clpp | ACTGTGCACACGGACAGTAG | CTGGATCTTTGGCCGGACTT |
| Lonp1 | GGAGAAGACCATTGCGGCTA | AGATGTCGCGGTAGTGTTCC |
| Hspa9 | CTGTCCAGCCGCCATGATAA | GAAGGGGTAGTTCTGGCACC |
| CLPP | GTTGCCAGCCTTGTTATCGC | TGCATCGTGTCGTAGATGGC |
| HSP60 | CCGCCCCGCAGAAATG | AGCATTAAGGCTCGGGCATC |
| HSP70 | GACTGCTTTTTGGGGCAGTG | AAGCAGGTACCAACTCTGGC |
| LONP1 | TGGCTACGTGCGACTGTG | GGAAACACATCGGGGATCGT |
| Mt-Nd1 | GGGTTGGGGCGATAATAAAT | ATGGCCTTCCTCACCCTAGT |
| Cox5a | TTGATGCCTGGGAGTTGCGTAAAG | ACAACCTCCAAGATGCGAACAGC |
| Sdhc | GCAAGAACCAAGACCGTCACTCC | TCCGACACTTGATGTGGGACCTAG |
| Ndufb5 | CCGTTGCCTACCCTACTTCC | GGAGCGACAGTCTTCGGAAA |
