# Supplementary material for: The mitochondrial UPR induced by ATF5 attenuates intervertebral disc degeneration via cooperating with mitophagy
Source: Cell Biol Toxicol. 2024 Mar 13;40(1):16. doi: 10.1007/s10565-024-09854-9 (PMC10933207; doi:10.1007/s10565-024-09854-9)
Supplement: Supplementary file 8 — Supplementary file8 (DOCX 28 KB) [file 10565_2024_9854_MOESM8_ESM.docx]

Table 4 Antibody information

| **Antibodies human (mouse or rat)** | **Source** | **Item No** |
| --- | --- | --- |
| Anti-HSP70 (Hspa1a) | Proteintech | Cat No. 10995-1-AP |
| Anti-LONP1 (Lonp1) | Proteintech | Cat. No. 15440-1-AP |
| Anti-CLPP (Clpp) | Proteintech | Cat. No. 15698-1-AP |
| Anti-HSP60 (Hspd1) | Proteintech | Cat No. 15282-1-AP |
| Anti-Bcl-2 | Proteintech | Cat No. 26593-1-AP |
| Anti-Bax | Proteintech | Cat No. 50599-2-Ig |
| Anti-PINK1 (Pink1) | Proteintech | Cat. No. 23274-1-AP |
| Anti-Sqstm1 | Proteintech | Cat. No. 18420-1-AP |
| Anti-Aggrecan | Proteintech | Cat. No. 13880-1-AP |
| Anti-Atf5 | Proteintech | Cat No. 67066-1-Ig |
| Anti-Tubulin | Proteintech | Cat No. 66031-1-Ig |
| Anti-Cleaved-Caspase 3 | Cell Signaling Technology | Cat. No. #9661 |
| Anti-Cleaved-Caspase 9 | Cell Signaling Technology | Cat. No. #9507 |
| Anti-Parkin | Abcam | Cat. No. ab77924 |
| Anti-Nqo1 | Abcam | Cat. No. ab80588 |
| Anti-Hmox1 | Abcam | Cat. No. ab13248 |
| Anti-Collagen Ⅱ | Abcam | Cat. No. ab188570 |
| Anti-Collagen Ⅰ | Abcam | Cat. No. ab260043 |
| Anti-TOMM20 | Abcam | Cat. No. ab186735 |
| Anti-LC3 | Novus | Cat. No. NB100-2220 |
| Anti-β-actin | Beyotime Bitotechnology | Cat. No. AF0003 |
